# Impact of Regional Vein Thrombosis in Patients with *Klebsiella pneumoniae* Liver Abscess

**DOI:** 10.1371/journal.pone.0140129

**Published:** 2015-10-07

**Authors:** James S. Molton, Yen Lin Chee, Tiffany P. Hennedige, Sudhakar K. Venkatesh, Sophia Archuleta

**Affiliations:** 1 University Medicine Cluster, National University Health System, Singapore; 2 Department of Medicine, Yong Loo Lin School of Medicine, National University of Singapore, Singapore; 3 Department of Hematology-Oncology, National University Health System, Singapore; 4 Department of Diagnostic Imaging, National University Health System, Singapore; 5 Department of Radiology, Mayo Clinic, Rochester, Minnesota, United States of America; University of North Dakota, UNITED STATES

## Abstract

Klebsiella liver abscess (KLA) is an emerging infection in Asia caused by hypermucoviscous strains of *Klebsiella pneumoniae*. It is associated with thrombophlebitis of portal and hepatic veins. The natural history and role of anticoagulation for this regional thrombophlebitis is unclear. In a retrospective study of 169 subjects with KLA over 7 years, thrombophlebitis was identified in 53/169 (31.4%). Only 1 received therapeutic anticoagulation. Despite this 30/49 (73.2%) of those with follow up scan available showed improvement or recanalization (mean duration between scans 44 days). Abscess resolution was associated with improvement in thrombophlebitis.

## Introduction

Klebsiella liver abscess (KLA) is common in Asia, representing a distinct syndrome caused by strains of *Klebsiella pneumoniae* bearing the hypermucoviscous phenotype mediated by the K1 or K2 capsule type [1]. KLA differs from other forms of pyogenic liver abscess in that it affects individuals without biliary disease and is strongly associated with diabetes mellitus [2]. The syndrome is notable for an increased incidence of metastatic infection to lung, eye or brain [1]. There are distinct radiologic features [3–7]. Whereas other pyogenic liver abscesses are often multiple and unilocular, KLA are more frequently single and multilocular. KLA may be associated with thrombophlebitis of hepatic or portal veins, although reported incidence varies. We have previously reported regional thrombophlebitis in up to one third of cases of KLA, as compared to only 5% in other causes of liver abscess [7]. Another report identified thrombophlebitis rates of 42% in pyogenic liver abscess but did not differentiate by causative organism [6]. Others reviewing radiologic appearances of KLA have not reported thrombophlebitis rates [3–5]. Septic thrombophlebitis has been identified as a risk factor for haematogenous spread of infection [7]. However optimal management is undefined. Anticoagulation of portal vein thrombosis secondary to liver abscess is controversial. A recent study showed spontaneous resolution of pylephlebitis in two thirds of patients with KLA treated with antibiotics alone [8]. To date no study has addressed management of hepatic vein thrombosis in KLA.

## Materials and Methods

### Ethics Statement

The relevant institutional review board—National Healthcare Group (NHG) Domain Specific Review Board (DSRB)—approved this study and waived the need for written informed consent from study participants.

A retrospective analysis was conducted of all patients presenting to our institution between May 2004 and December 2011 with a liver abscess diagnosed on contrast-enhanced CT scan, with *K*. *pneumoniae* identified on blood or liver abscess fluid cultures. Demographic, clinical and radiologic data were extracted from the medical records. Radiology reports were manually reviewed for description of regional thrombosis (defined as thrombosis or thrombophlebitis in the inferior vena cava (IVC), hepatic veins or tributaries, or the portal vein). The following variables were investigated for influence on risk of developing regional thrombosis: sex (male/female), presence of diabetes (obtained from case record), mobility status (immobile defined as wheelchair bound or bed bound), presence of bacteraemia (defined as blood culture positive for *K*. *pneumoniae*), and by whether the abscess was septated or multifocal on imaging. The following variables were investigated for risk of progression of the thrombus: diabetes, mobility, location of thrombus (hepatic circulation (including IVC) versus portal vein), whether therapeutic anticoagulation was given, whether the abscess was drained and whether the abscess had resolved at follow up scan. Categorical variables were compared using Fisher’s exact test and continuous variables were compared using t test.

## Results

169 patients were identified. The median age was 58 years. 71% were male, 55% were diabetic, and 22.5% required ICU admission. 66.3% were bacteraemic with *K*. *pneumonia*. 66.9% of liver abscesses were in the right lobe, 26.6% in the left lobe and 2.4% in the caudate lobe. The remainder were in more than one lobe. The mean abscess maximal diameter was 6.7cm. 63.3% were percutaneously radiologically drained, 13.6% surgically drained, and 23.1% required no drainage. Appropriate antibiotics were given in all cases.

Thrombophlebitis was identified on CT in 53 patients (31.4%), of which 3 were affecting portal vein, 49 hepatic vein and one inferior vena cava (IVC) (Table 1).

**Table 1:** Location of thrombosis.

**Inferior vena cava**	1
**Right hepatic vein or tributary**	28
**Middle hepatic vein or tributary**	13
**Left hepatic vein or tributary**	12
**Intrahepatic portal vein**	3

Location of thrombosis (n = 53). Note thrombosis may be in more than one site

Three subjects had clots extending to the IVC. Thrombosis was unrelated to age, gender, diabetes, immobility (wheelchair or bedbound), presence of bacteraemia, abscess size, or whether the abscess was multiple or loculated (Table 2). Nine patients had concomitant pneumonia which may represent septic metastatic infection.

**Table 2:** Risk factors for thrombosis.

	Thrombosis (n = 53)	No thrombosis (n = 116)	p-value
**Gender (male)**	37 (69.8%)	83 (71.6%)	0.86
**Mean Age (years)**	56.77	58.65	0.43
**Diabetes mellitus**	32 (60.4%)	61 (52.6%)	0.41
**Immobility**	2 (3.8%)	2 (1.7%)	0.59
**Bacteraemia**	39 (73.6%)	73 (62.9%)	0.22
**Mean abscess size (cm)**	6.7	6.6	0.83
**Septation/Loculation**	52 (98.1%)	110 (94.8%)	0.44
**Multifocal abscess**	5 (9.4%)	11 (9.5%)	1.00

41 patients with thrombosis had a follow up CT scan for comparison.The mean interval time between scans was 44 days. 30 (73.2%) revealed improvement or complete recanalization, 9 (22.0%) showed interval stability and 2 (4.9%) showed extension of the thromboses (Table 3). The two who showed extension had no complications and were cured clinically. Recanalization of the affected vein was strongly associated with complete abscess resolution. Of the 30 patients with recanalization or reduction in size of thrombus, 11 (36.7%) showed complete abscess resolution, compared to none in the group that did not show improvement of the thrombus (p = 0.02). Diabetes, immobility, location of thrombus (hepatic versus portal) and drainage of the abscess were not associated with thrombus resolution. Scans were assessed for radiologic evidence of thrombotic complications such as ascites, splenomegaly, collaterals and cavernous transformation. Only 3 cases of ascites were identified, all in patients with hepatic vein thrombosis. At follow up scan the ascites had resolved in two cases and improved in the other, without anticoagulation. No other radiologic complications were identified.

**Table 3:** Radiologic outcomes for those individuals with a follow up scan available.

	Improvement or complete recanalization (n = 30)	Stable, or thrombus extension (n = 11)	p-value
**Diabetes mellitus**	16 (53.3%)	5 (45.5%)	0.73
**Immobility**	0	1 (9.1%)	0.27
**Hepatic circulation thrombus**	28 (93.3%)	11 (100%)	1.00
**Anticoagulation given**	0	1 (9.1%)	0.27
**Abscess drained**	25 (83.3%)	9 (81.8%)	1.00
**Abscess resolution at follow up scan**	11 (36.7%)	0	0.02

In terms of clinical outcomes, there was no significant difference between rates of intensive care unit (ICU) admission, mean length of hospitalization or mortality between those with and without thrombosis. Six patients in the study died (3.6%). In all cases the cause of death was sepsis. Of these, three had hepatic vein thrombosis. Two had a follow up scan of which one showed no recanalization and one showed improvement.

Of the 53 patients with septic thrombophlebitis, only one received therapeutic anticoagulation. This patient had a 13.3cm right lobe liver abscess with middle and left hepatic vein thrombosis extending to the IVC. The abscess was radiologically drained and *K*. *pneumoniae* was isolated from blood and abscess fluid. The patient developed metastatic infection to the lungs with pneumonia requiring intubation, but no pulmonary embolus was identified. Following 28 days of therapeutic anticoagulation with low molecular weight heparin (LMWH), the patient was well and an ultrasound showed complete abscess resolution. LMWH therapy was complicated by hematuria. It is worth noting that of the three patients with pylephlebitis none were anticoagulated. Two had follow up scans available, of which one showed complete recanalization and one showed improvement. All three were clinically cured without complications. Incidentally there were two patients without septic thrombophlebitis who received therapeutic anticoagulation for other indications; one for lower limb deep vein thrombosis (DVT) and one for pulmonary embolism (PE). The subject with DVT developed a gastrointestinal bleed as a complication of anticoagulation. 6 patients in the total cohort received prophylactic anticoagulation with LMWH, 3 in the group with thrombosis and 3 in the group without thrombosis.

## Discussion

This study identified thrombophlebitis in nearly one third of patients with KLA. The natural history of these thrombosis has not been described previously and the role of anticoagulation has not been defined in this population.We found little clinical consequence of septic thromboses of portal vein or hepatic veins. The one patient with PE had no evidence of thrombophlebitis in the liver.

It would appear that in KLA the vast majority of hepatic vein septic thromboses resolve spontaneously without anticoagulation. The thromboses improved as the abscess resolved, suggesting management should be targeted at the underlying abscess. Although too few to draw any real conclusions it is worth noting that the 3 portal vein thromboses in this series resolved spontaneously, which is in keeping with the recent observation of high rates of spontaneous recanalization of portal vein thromboses in KLA [8].

Whilst septic thrombophlebitis at presentation has been associated with metastatic infection, most of this metastasis occurred prior to presentation [7]. Targeting the thrombus once antibiotics have been initiated does not appear to be necessary, and furthermore we have demonstrated that therapeutic anticoagulation is not without risk. While we cannot rule out a role for anticoagulation in a subset of patients, the priority should be on early diagnosis with prompt drainage of the abscess and initiation of appropriate antibiotics.

## Supporting Information

S1 Dataset Dataset file containing all study data. (XLS)
